# Temporal binding of social events less pronounced in individuals with Autism Spectrum Disorder

**DOI:** 10.1038/s41598-022-19309-y

**Published:** 2022-09-01

**Authors:** David H. V. Vogel, Mathis Jording, Carolin Esser, Amelie Conrad, Peter H. Weiss, Kai Vogeley

**Affiliations:** 1 Institute of Neuroscience and Medicine, Cognitive Neuroscience (INM3), Research Center Juelich, Jülich, Germany; 2 Faculty of Medicine and University Hospital Cologne, Department of Psychiatry, University of Cologne, Cologne, Germany; 3 Faculty of Medicine and University Hospital Cologne, Department of Neurology, University of Cologne, Cologne, Germany

**Keywords:** Cognitive control, Autism spectrum disorders, Human behaviour, Sensorimotor processing, Social behaviour

## Abstract

Differences in predictive processing are considered amongst the prime candidates for mechanisms underlying different symptoms of autism spectrum disorder (ASD). A particularly valuable paradigm to investigate these processes is temporal binding (TB) assessed through time estimation tasks. In this study, we report on two separate experiments using a TB task designed to assess the influence of top-down social information on action event related TB. Both experiments were performed with a group of individuals diagnosed with ASD and a matched group without ASD. The results replicate earlier findings on a pronounced social hyperbinding for social action-event sequences and extend them to persons with ASD. Hyperbinding however, is less pronounced in the group with ASD as compared to the group without ASD. We interpret our results as indicative of a reduced predictive processing during social interaction. This reduction most likely results from differences in the integration of top-down social information into action-event monitoring. We speculate that this corresponds to differences in mentalizing processes in ASD.

## Introduction

Diagnostic manuals define Autism Spectrum Disorder (ASD) as comprising of symptoms within two broader categories. These categories cover (i) “persistent deficits in social communication and social interaction” and (ii) “restricted, repetitive patterns of behavior, interests, or activities”^1^. As these two categories differ substantially, research continually attempts at describing psychological or (neuro-) physiological processes that might account for symptoms from both categories.

From amongst these, the concept of variations in predictive processing has gained considerable traction^2,3^. The proposal involves so-called hypo-priors presumably defining the predictive processes of persons with ASD. The theory assumes that the perceptual input of individuals with ASD consist of more detailed perceptual input than of those without ASD. The higher perceptual detail of hypo-priors presumably leads to a comparatively slower information processing speed as more information needs to be weighed^4^. When faced with complex situations, such as in most social contexts, this leads to asynchronies^5^ and from a third person perspective to “deficits in social communication and social interaction”^1^. The reported and observed “restricted, repetitive patterns of behavior, interests, or activities” can then be understood as compensatory behaviors and preferences directed at reducing situational complexity or at withdrawing from the situation all together^6–8^.

The prediction and processing of percepts generally relates to the integration of multisensory information—i.e., the processing of events entailing perceptual input from multiple sensory sources^9,10^. Multisensory integration is thought to depend on a so-called temporal binding window^11,12^. This window describes the interval that may lie between two (multisensory) events for them to be perceived as belonging to a perceptual unit. Non-surprisingly, theory suggests that binding works differently in individuals with ASD^13^. Correspondingly, the temporal binding window is assumed to be larger in ASD^14–17^ resulting in a higher information load and disconnectedness of perceptual events which are usually processed as units.

The temporal binding effect (TB) presents a particularly valuable paradigm to assess predictive processes behaviorally^18–20^. TB describes the observation of a temporal misperception of related events^21^ or of the duration between two such events^22,23^. Most often, TB occurs in the context of intentional action and refers to a relative lower estimate of a duration between an action and its consequence as compared to the same duration between two, e.g., observed events. In this context, TB refers to the “systemic bias”^23^ in time estimates between action-event-sequences and event-event-sequences not involving an action. The method has also been referred to as “temporal magnitude estimation”^24,25^.

Most commonly, TB is reported in the context of the Sense of Agency (SoA). SoA stands for the feeling of being in control of one’s actions and consequently their outcomes. TB poses a measurable correlate to SoA—then referred to as “intentional binding” (for review see^18^). Recent investigation, however, demonstrated that this intentional binding is a particular subtype of TB. The effect seems to appear whenever two events are perceived as a perceptual and/or causal unit^20,26^. SoA related action-event-sequences are thought to be particularly strong units.

In experimental contexts involving high contingency, TB particularly relies on predictive processes^27,28^. The prediction is thought to be more reliable the more information about the involved events is available^29^.

Recent insight makes TB an interesting avenue of investigation for ASD. For one, the effect is clearly understudied for a syndrome implicated to be caused by differences in predictive processes. So far, only two studies have investigated TB and ASD with different methodology as well as diverging results. Sperduti et al.^30^ investigated TB using auditory, visual, and multimodal (combined visual and auditory) stimuli as action dependent events. They found an absence of TB for visual stimuli and a decreased TB for the other two modalities in persons with ASD compared to a control population without ASD. Finnemann et al.^31^ recently did not confirm significant differences in TB between a group of participants with ASD and a group of participants without ASD.

The studies’ seemingly conflicting findings are difficult to interpret. The experiment by Sperduti et al.^30^ involves a comparatively small sample size with statistically small effects. Finnemann et al.^31^ on the other hand investigated TB with a more conservative method, not involving a time estimation task, but a Libet Clock with synchrony judgements (for review see^18^). Furthermore, both studies involve events of low complexity (i.e., auditory beeps and visual flashes). While Finnemann et al.^31^ correctly deduce from their results that predictive processes are not detectably different between people with and without ASD, their findings raise the question whether more complex, e.g., social events, might produce the same finding.

Social events are interesting for at least three reasons. First, difficulties with and during social situations is a primary concern for many persons with ASD. Second, social events enhance TB^32^. TB seems to be stronger for joint actions with other^33,34^, similarly for a partner’s actions as compared to one’s own actions^35^, and stronger during leadership as opposed to follower situations^36,37^. In the context of these investigations, TB again relates to the sense of agency (SoA) and of joint agency (SoJA) with stronger TB indicating stronger SoA^18^.

Lastly, belief in the presence of an interaction partner poses a strong influence on predictive mechanisms. Arguably, this influence is mediated by additional processes recruited during activities involving prior socio-cognitive top-down information^38,39^. A multitude of studies has demonstrated that several tasks that require to take another person’s inner experience or behavior into account are performed differently by individuals with ASD^39–43^. For example, mentalizing has been assumed as a mechanism by which persons without ASD increase perceived contingency between events during social interaction and hence strengthen predictive processing^44^. Mentalizing capacities are often decreased in individuals with ASD^45–47^. Hence, it is plausible that its influence on perceptual prediction, and correspondingly TB, are also diminished in individuals with ASD.

To investigate this hypothesis, we employed a paradigm by Vogel et al.^32^ designed to specifically address the question of TB in social contexts. The recent study found that the estimation of a time interval between a button press and a movement on a computer screen is influenced by the presumed nature of the observed movement: Duration judgements were shorter whenever participants assumed that a person responded to one’s own action as opposed to a physical effector (computer). In other words, TB was stronger whenever the moving stimulus was bearing social information.

In the following two experiments, we will first describe and discuss a replication of the experiment by Vogel et al.^32^, as well as the extension to a group of individuals with ASD. We secondly report a variation of the experiment to better examine the influence of belief in social interaction on TB for both individuals with and without ASD.

## General methods

This study consisted of two experiments, both involving participants with and without ASD. Experiment #1 explicitly served as replication and extension of a previous experiment^32^ investigating differences in human–human interaction and human–computer interaction. Experiment #2 was designed after analysis of the results from Experiment #1 to focus on a particular aspect of the results of interest for ASD.

Both experiments made use of a cover story involving a confederate. Involvement of the confederate was performed as described by Vogel et al.^32^. Participants were led to believe they were performing the experiment together with another person. After having taken a seat in the room where the experiment took place, but prior to starting the experiment, participants were introduced to another person of the same gender and similar age as their partner for the study. In fact, the person introduced was a confederate of the experimenter and not active during the experiment, and the experiment was entirely computer-controlled. The instructions emphasized its interactive nature where possible by employing repeated mentions of the interaction partner and the repeated use of the words “interactive”, “together”, and “cooperation”. Participants thought they had been randomly assigned the “active” part in the experiment, meaning that they were to be giving orders to their partner via their computer by pressing either the left or the right arrow key. The assumed interaction partner would then have to act on the order as quickly as possible by either diverging their gaze to the left or to the right according to the pressed key.

Participants were made to believe that their partner was sitting in an adjacent room in front of an eye-tracker. We showed participants an eye-tracker and introduced them to its function. We told participants that the eye-tracker in the adjacent room was to measure the partner’s eye movements and depict them on the participant’s screen in real time. Participants were instructed to estimate their partner’s reaction time. We encouraged participants to use both keys as to avoid bias to one side.

### Experiment #1

#### Methods for Experiment #1

##### Stimuli and apparatus

**Figure 1:**
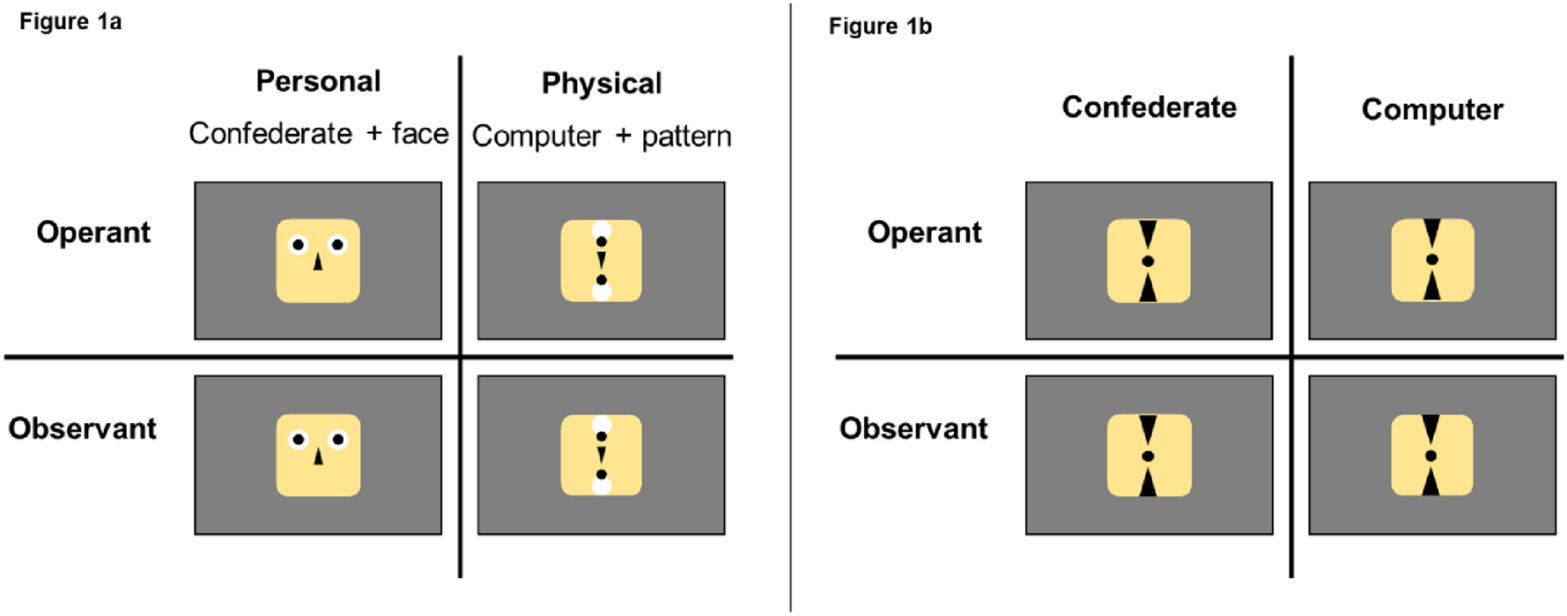
Conditions for Experiment #1 and Experiment #2: (**a**) Factors for *Experiment #1* are depicted on the left (Vogel et al., 2021). Face stimulus and belief in a Confederate, and a pattern stimulus and belief in an interaction with the computer were combined congruently. Combinations (personal vs. physical) were compared across an operant and an observant condition (operant-personal, operant-physical, observant-personal, operant-physical). (**b**) Factors for *Experiment #2* are depicted on the right. Participants saw the identical stimulus across all trials. The cover story was systematically varied across experimental blocks. The resulting combinations during blocks were operant-confederate, observant-confederate, operant-computer, and observant-computer.

Stimulus and apparatus were used according to Vogel et al.^32^. Two stimuli, combined with the cover story served as representation of the interaction partner (Fig. 1a). A standardized face constructed from geometric shapes and a corresponding pattern stimulus made up of the identical proponents were presented to represent an interaction with a confederate or with the computer, respectively. In other words, when participants were presented with the face stimulus they were told to be interacting with a person, while, when presented with the pattern, they were told to be performing tasks alone, on their computer. Both stimuli were of identical size and presented at roughly 8 degrees visual angle.

The experimental paradigm was programed and performed in PsychoPy2^48^. Stimuli were presented on a 22-inch computer screen (resolution 1680 × 1050 pixels) against a standard grey background. Viewing distance was approximately 70 cm. A standard computer keyboard and mouse were used for participants’ responses.

##### Procedure

A 2 × 2 × 2 factorial design with the factors agency (observant vs. operant), partner (physical vs. personal), and interval (400 ms vs. 700 ms) constituted the paradigm. The experiment consisted of four blocked conditions of 60 trials each. Each block started with written and standardized oral instructions to the participant. Blocks contained a combination of the factors agency and partner, resulting in four blocks (observant-physical, observant-personal, operant-physical, operant-personal) (Fig. 1). The different intervals were randomized within blocks. We counterbalanced block order across participants. Participants performed 60 trials per block resulting in 30 trials per condition.

In all conditions, the respective stimulus was presented at the start of each trial. For observant blocks, participants were instructed to pay attention to their computer screens. 2.5 s to 3.5 s after the trial had started, a white arrow appeared below the stimulus either pointing to the right or to the left. After either 400 ms or 700 ms (factor interval), the stimulus changed its configuration; in the case of the face stimulus, the black dots representing the stimulus’ pupils moved to the right, or the left, depending on the direction indicated by the arrow; in the case of the pattern stimulus, the two black dots on the vertical axis moved to the right, or the left, depending on the direction indicated by the arrow. For operant blocks, participants were instructed to press either the left or the right arrow key on their keyboard. After either 400 ms or 700 ms (factor interval), the stimulus changed its configuration; in the case of the face stimulus, the black dots representing the stimulus’ pupils moved to the right, or the left, depending on the direction of the pressed arrow key; in the case of the pattern stimulus, the two black dots on the vertical axis moved to the right, or the left, depending on the direction of the pressed key.

Participants were instructed to press a key at a time of their choosing. No stimulus change or appearance occurred before participants pressed a key. Although the lack of a sufficient preparation time may interfere with trial performance^49^, to guarantee the impression of a voluntary movement promoting TB^21,50^, we provided neither an upper time limit nor a lower minimal preparation time for participants to perform the key press.

The subsequent time estimation task was based on the established procedure first used by Engbert et al.^22,23^. For all trials, participants estimated the delay between the initial event (key press or arrow appearance) and the subsequent event (dot movement). For these time estimates, a visual analogue scale (VAS) appeared on the computer screen. The bottom anchor of the VAS was 0 ms representing the perception of immediacy. The top anchor was 1000 ms. Instructions told participants to remember that 1 s contained 1000 ms and that the line of the VAS hence represented one second. The VAS appeared without any marked duration. After clicking on the VAS with their computer mouse a blue cursor appeared above the scale and below the scale appeared the numerical estimate. Participants could then adjust their estimate by using their mouse to move the cursor along the VAS. Participants did not perform any practice trials prior to the experiment. Participants naively judged the given durations.

The experimental design was supposed to create a high contingency environment. For all physical conditions (both operant, observant), the pattern stimulus was presented, and participants were made to believe they were interacting with a computer algorithm. For all personal conditions (both operant, observant), the face stimulus was presented, and participants were made to believe they were interacting with a confederate. For the operant, personal block, participants were instructed to be giving orders to their human partner (confederate), allegedly seated in an adjacent room. For the observant, personal block participants thought they were watching as their human partners responded to stimuli given by the computer. In line with the cover story, we told participants that their interaction partner (confederate) was controlling the face stimulus’ eyes by their own gaze movements through an eye-tracker which were depicted on the participants computer screen in real time.

**Figure 2:**
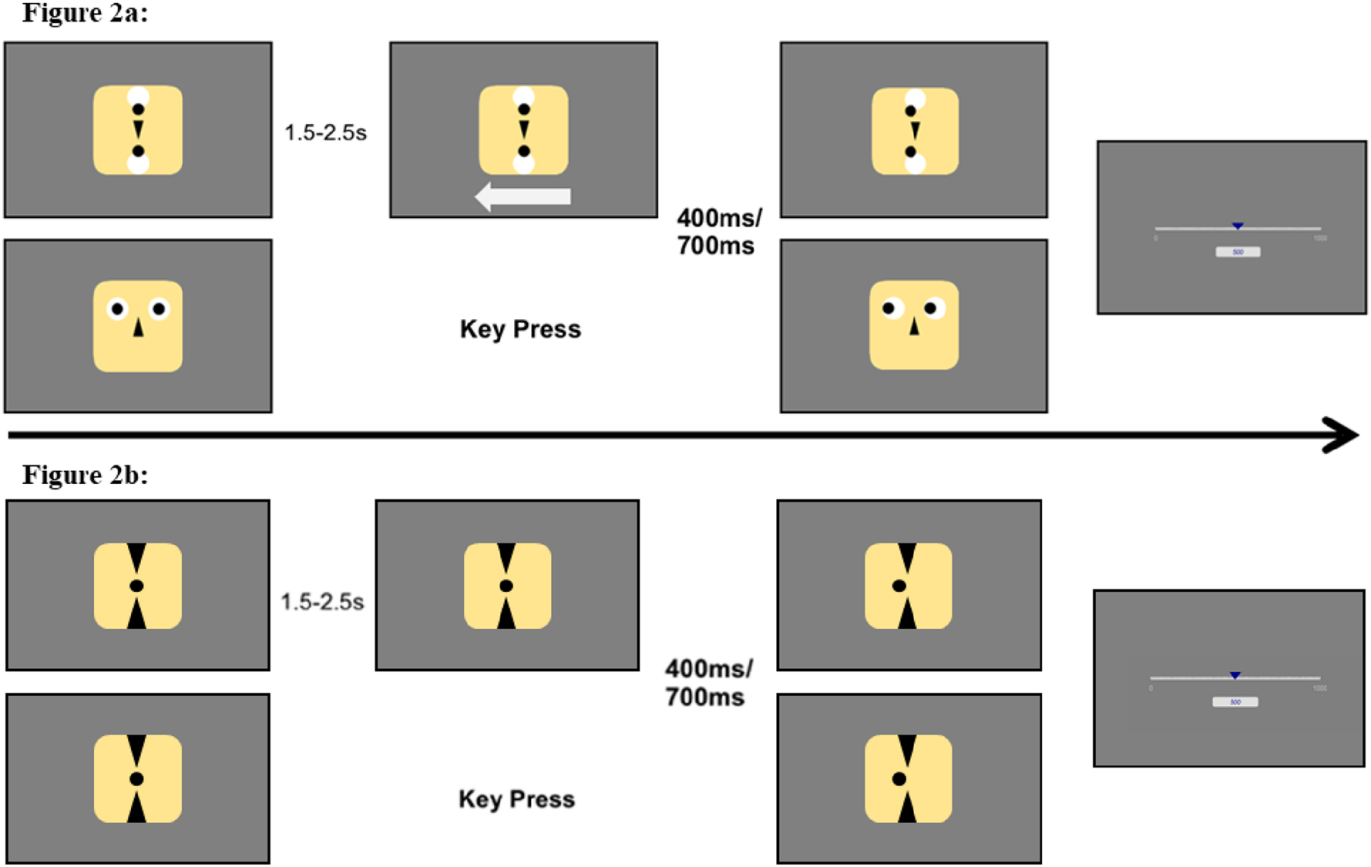
Trial Event Structure. (**a**) shows the set-up of Experiment #1 for the physical-observant (top row) and the personal-operant (bottom row) trials. (**b**) shows the set up of Experiment #2 for the observant (top row) and the operant trials. *Top row* Trials started with the depiction of the respective stimulus. For observant conditions an arrow appeared after 1.5–2.5 s to indicate movement direction and to serve as the start event for the following duration judgement. After either 400 ms or 700 ms the stimulus moved its dot(s)/eyes to the left or right depending on indicated direction. Lastly, participants estimated the duration between arrow presentation and stimulus movement using a visual analogue scale. *Bottom Row* Trials started with the depiction of the respective stimulus. For operant conditions, participants freely pressed one of two buttons indicating a movement direction and to serve as the start event for the following duration judgement. After either 400 ms or 700 ms the stimulus moved its dots/eyes to the left or right depending on indicated direction. Lastly, participants estimated the duration between key press and stimulus movement using a visual analogue scale.

For all physical conditions, instructions were identical to the personal conditions, with the difference that during operant, physical conditions participants would be giving orders to the computer and during physical-observant conditions they would be watching two stimuli presented by the computer. We instructed participants that the durations needed by the computer to initiate dot movements were programmed to be reflecting human reaction times. Figure 2a depicts the trial event structure of Experiment 1.

We introduced two further cues to improve the credibility of the cover story. Prior to the first trail of personal blocks, participants saw a notification reading “Connecting to Partner Computer…” on their screens. Additionally, a mock phone call was placed to the alleged second test room to increase believability. Furthermore, every 1/6 trials were fail-trials to increase credibility. For fail trials, the stimulus’ dots moved the opposite direction than indicated or instructed. Participants were told that errors by their partners were to be expected and that errors during blocks without the partner were necessary for later statistical analysis.

After the experiment, we debriefed participants in a structured interview. Interviews primarily were conducted to guarantee that participants had believed the cover story and clearly understood the instructions. Any participant indicating sufficient doubt or disbelieve in the reality of the cover story was excluded from later analysis. If it became apparent that a participant had not understood the instructions, they were excluded from later analysis.

To compute required sample sizes, we performed an a priori power analysis for t-tests on differences between means of matched pairs. Given an alpha error probability of 0.05, a desired power of 0.85, and using Cohen’s dz = 0.68 based on the effect sizes from the original experiment by Vogel et al.^32^, we calculated a minimum sample size per group at n = 22 in G*Power^51^. Data analysis was conducted using SPSS 25^52^ and the R based^53^ software jamovi^54^.

##### Participants

We recruited 59 participants (23 females, 36 males). 11 participants (5 identifying as female, 6 as males), were excluded: Data from two persons had to be omitted due to technical difficulties; nine participants did not believe the cover story. Of the remaining 48 participants included in the experiment, 24 participants [9 identifying as female, 15 as males; mean age 42.46 years (SD = 8.79)] had been diagnosed with Asperger Syndrome (ASD) at the Autism Outpatient Clinic at the Department of Psychiatry, University Hospital Cologne, Germany; and a confirmed IQ above 80. The second group consisted of 24 typically developed (TD) participants (9 identifying as female, 15 as males; mean age 42.33 years (SD = 8.83)). Participants were matched by age and gender between groups. We obtained Autisms Quotient (AQ)^55^ scores from all participants.

All participants reported normal or corrected-to-normal vision and hearing. Participants in the TD group were only included if they reported no current psychiatric or neurological diagnosis and denied the use of neuropsychiatric or psychoactive drugs for the two weeks prior to inclusion. Participants within the ASD group were included if they reported no current psychiatric or neurological diagnosis except for ADHD and MDD as the arguably most common psychiatric co-morbidities of ASD. However, none of the participants included into this experiment reported any co-morbid diagnoses.

#### Results for Experiment #1

Independent sample t-tests were conducted to analyze differences in AQ scores. There was a significant difference between the AQ scores for the ASD group (mean = 38.88, SD = 5.79) and the TD group (M = 18.82, SD = 5.73); t(46) = 12.52, p < 0.000, with results ranging from 24 to 47 for the ASD group, and from 10 to 29 for the TD group.

VAS are prone to anchor bias depending on their bottom and top anchors (e.g.,^56–58^). The bias occurs when the instructions for the bottom and top value are systematically misinterpreted or interpreted differently between participants. Anchors are regularly used by individuals when judging durations^59–61^. To rule out interference of bottom and top anchors—i.e., a misinterpretation, false conceptualization, or misperception of immediacy and/or 1 s—we performed one-sided one-sample t-tests (Student’s T-tests for normal distributions, Wilcoxon rank sum tests for non-normally distributed data) of individual estimates against 0 ms and against 1000 ms to ensure sufficient deviation from the VAS anchors in all individuals. All participants showed significant (p < 0.05) divergence from 0 and from 1000.

To account for variance caused by interindividual anchoring we analyzed time estimates in a linear mixed effects model as recommended for repeated measures designs^62^ with random intercepts for participants. Outliers in the main results plots relate to within-participants anchoring of participants estimating time at the top end of the VAS.

Visual inspection of residual plots did not reveal any obvious deviations from normality or homoscedasticity. We compared trials from the different conditions based on the factors partner, agency, interval. A significant effect reflecting a lower estimate of one level of a condition as compared to the other was interpreted as TB.

Results confirm our initial hypothesis concerning significant differences for the three main factors: Agency (Observant (mean (averaged across all other conditions) ± Standard Deviation): 419 ms ± 251 ms, Operant: 402 ± 259 ms; mean difference (M) = − 16.58, Standard Error (SE) = 3.09, t = − 5.367, p < 0.001), partner (Physical: 422 ms ± 255 ms, Personal: 399 ms ± 254 ms; M = − 22.71, SE = 3.09, t = − 7.351, p < 0.001), and interval (400 ms Interval:: 362 ms ± 235 ms, 700 ms Interval: 459 ms ± 264 ms; M = 97.77, SE = 3.09, t = 31.645, p < 0.001). These results indicate TB for operant as compared to observant trials, for personal as compared to physical trials, and shorter time estimates for 400 ms trials as compared to 700 ms trials. The factor group did not reach statistical significance, showing no discernable general difference in time estimation for ASD versus TD. Although we did not use any catch trials, participants were generally able to discriminate reliably between 400 and 700 ms intervals. We therefore assume that participants payed sufficient attention to the task.

**Figure 3:**
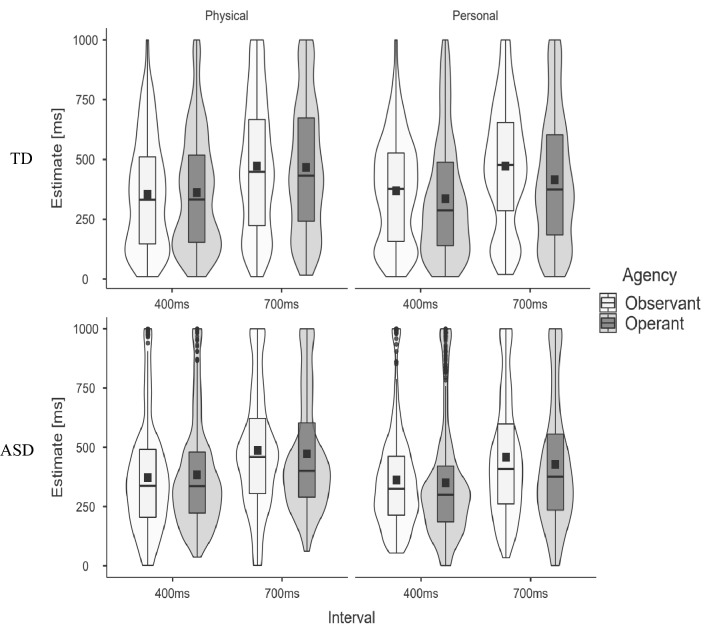
Results for Experiment #1. The top row depicts estimates from the TD group. The bottom row depicts estimates from the ASD group. Plots on the left depict estimates for physical conditions. Plots on the right depict estimates for personal conditions. Time estimates for 400 ms interval conditions are shown in the left columns; those for 700 ms interval conditions are shown in the right columns. Light grey indicates observant conditions, while dark grey indicates operant conditions. Outliers (depicted as black dots) correspond to individual participant’s estimates and reflect grouping around individual anchor points. Social hyperbinding is visible in the relatively lower estimates of operant trials as compared to observant trials for personal conditions. Hyperbinding is lower for the ASD group.

We found a significant interaction between agency and partner (M = − 33.24, SE = 6.18, t = − 5.380, p < 0.001) replicating social hyperbinding for this data set. In other words, the relative lower mean estimates for operant trials was further pronounced for social conditions (see Fig. 3). The interactions between partner and interval (M = − 17.23, SE = 6.18, t = − 2.789, p = 0.005), and agency and interval (M = − 19.83, SE = 6.18, t = -3.209, p = 0.001) also reached statistical significance. These interactions reveal that during trials involving larger intervals, differences between estimates for operant versus observant trials and for personal versus physical trials were larger. This interval effect replicates our previous findings, which were due to a relative floor effect for the smaller time estimates^32^. As 400 ms are closer to physiological reaction times than 700 ms, the smaller intervals offer more leeway for smaller judgements. Conversely, the larger intervals can be judged lower. This in turn may have caused participants to provide smaller judgements for operant-personal conditions for 700 ms intervals.

The three-way interaction between agency, interval, and partner did not reach statistical significance (M = − 1.58, SE = 12.36, t = − 0.128, p = 0.898).

Concerning group differences, the interaction between the factors partner and group reached significance (M = − 12.96, SE = 6.18, t = − 2.097, p = 0.036) indicating that the difference of reported estimates between the conditions Physical and Personal was larger for the TD group than for the ASD group.

Importantly, the three-way-interaction between agency, partner, and group reached significance (M = 26.62, SE = 12.36, t = 2.154, p = 0.031). This interaction reflects a more pronounced hyperbinding (i.e., the interaction between partner and agency) in the TD group (see Fig. 3).

No other interaction reached the significance threshold. We performed post-hoc analyses on additional variables of interest. These included a correlation of the effect of interest with autistic trait scores as measured by the AQ and an analysis of within-subjects variance (see Supplement).

We did not find a significant correlation between autistic traits and the hyperbinding effect (Table ST1.2a/b). As the lack of such an interaction in our data alone cannot provide sufficient evidence for either argument, future research should clarify whether a lower hyperbinding in ASD reflects a discrete criterium potentially reflecting a discrete condition, or, whether it runs along a continuum of autistic traits corresponding to a continuum of traits between persons with autism and persons without autism.

Our supplementary analyses for changes in participant-wise variance (Figs. SF1.3, 1.4; Table ST1.1) show an increase in variance from 400 ms intervals to 700 ms intervals. This finding if overall in line with the scalar expectancy theory of time perception^63^. However, they also suggest differences in variance between conditions and between groups (Fig. SF1.3). These differences in estimate precision do not precisely correspond to the main findings with variances in some cases increasing for conditions with lower estimates. We suggest that future investigation pay closer attention to intra-individual variance, arguably by employing a method more suitable to detect to assess general time perception capacities (see our discussion section for a more in-depth discussion of methodological limitations and further research directions).

The supplement further includes figures on the main effects containing further information, such as individual mean estimates and standard deviations (Figs. SF1.1 and 1.2).

#### Discussion for Experiment #1

The experiment served to detect the influence of social information on TB in a group of individuals with ASD as compared to a TD group. Our results overall replicate earlier findings by Vogel et al.^32^ for individuals without ASD. TB was primarily observable for conditions during which participants thought they were interacting with another person.

This finding strengthens the authors’ theory of a social hyperbinding during intentional actions directed at a human interaction partner. Notably, social hyperbinding was present in both groups. Overall, this finding demonstrates the importance of social information for TB for both diagnostic groups. The combination of cover story and face stimulus elicited TB for both groups, indicating their influence on predictive processing.

Although results reveal an overall group interaction in judgements between agency and partner, our analysis was unable to detect precisely whereby these interactions were determined. Effects estimates for the three-way interaction between agency, partner, and group suggest a positive mediation. In other words, the analysis indicates that the increase in TB detected for conditions with a confederate—social hyperbinding—is stronger in the group without autism. However, from this experiment we are unable to determine whether this effect was primarily driven by the targeted top-down information or by the bottom-up perceptual features of the two different stimuli.

To investigate this further, we designed a follow-up experiment directed more precisely at the top-down effects of experiment #1. The study design of experiment #1 presented a combination between stimulus material and a corresponding cover story. The combination of both manipulations in single trials might have influenced participant behavior, particularly in the ASD group. The bottom-up information presented with the stimulus might have a covert effect on the top-down belief in social interaction.

### Experiment #2

Our second experiment made changes to the initial investigation, augmenting the first experiment to better address top-down influences of belief in social interaction on TB in ASD. We simplified the study design by no longer relying on face stimuli, but using a single, non-face-like stimulus for all conditions (Fig. 1b). Thereby, the experiment no longer employed a double manipulation of top-down (confederate cover story) and bottom-up (stimulus) information but restricted itself to manipulating the cover story exclusively.

#### Methods for Experiment #2

##### Stimulus and apparatus

Experiment #2 was an alteration of Experiment #1. We dropped the two different stimuli and replaced them by a single non-face like stimulus (Fig. 1b). Accordingly, the purpose of experiment #2 was to elucidate the influence of the belief in the presence of another person by employing a focused version of Experiment #1. Stimuli were presented at a visual angle of 8 degrees.

##### Procedure

The procedure for experiment #2 was identical to that of Experiment #1. Other than the change in stimulus material, nothing was changed. To avoid confusion with results from Experiment #1, we relabeled the condition involving the cover story. This resulted in a 2 × 2 × 2 factorial design with the factors agency (observant vs. operant), story (computer vs. confederate), and interval (400 ms vs 700 ms).

The cover story was identical to that of Experiment #1, with the slight adjustment that we told participants that the human partner (confederate) was guiding the dot by their gaze movements through an eye-tracker. Figure 2b depicts the trial event structure of Experiment 2.

#### Participants

We recruited 52 participants (22 identifying as female, 30 as male). 7 participants (2 identifying as female, 5 as males) were excluded because they did not believe the cover story.

Of the remaining 45 participants included in the experiment, 23 participants [10 identifying as female, 13 as male; mean age 38.78 years (SD = 11.13)] had been diagnosed with Asperger Syndrome (ASD) at the Autism Outpatient Clinic at the Department of Psychiatry, University Hospital Cologne, Germany; and a confirmed IQ above 80. The second group consisted of 22 typically developed (TD) participants [10 identifying as female, 12 as male; mean age 38.09 years (SD = 12.05)]. Participants were matched by age and gender between groups. We obtained Autisms Quotient (AQ) scores from all participants.

All participants reported normal or corrected-to-normal vision and hearing. Participants in the TD group were only included if they reported no current psychiatric or neurological diagnosis and denied the use of neuropsychiatric or psychoactive drugs for the two weeks prior to inclusion. Participants within the ASD group were included if they reported no current psychiatric or neurological diagnosis except for ADHD and MDD. However, none of the participants included into this experiment reported co-morbid diagnoses.

#### Results for Experiment #2

Independent sample t-tests were conducted to analyze differences in AQ. There was a significant difference between the AQ scores for the ASD group (mean = 41.7, SD = 5.31) and the TD group (mean = 13.5, SD = 5.66) (t(43) = 16.96, p < 0.000).

We again performed one-sided one-sample t-test of individual estimates against 0 and against 1000 to guarantee sufficient deviation from the VAS anchors. Although one participant provided estimates particularly close to 0 ms (f.i., see Supplement SF2.2, SF2.4), all participants significantly diverged from both anchors.

Effects of experimental manipulations on time estimates again were analyzed using a linear mixed effects model as recommended for repeated measures designs^62^ with random intercepts for participants. Visual inspection of residual plots did not reveal any obvious deviations from normality or homoscedasticity. As in Experiment #1, we compared trials from the different conditions based on the factors partner, agency, interval. A significant effect reflecting a lower estimate of one level of a condition as compared to the other was interpreted as TB.

Results again confirmed our hypothesis on effects of and between the three main factors. Analysis revealed significant effects for agency (Observant (mean (averaged across all other conditions) ± Standard Deviation): 384 ms ± 219 ms, Operant: 346 ms ± 212 ms; mean difference (M) = − 37.83, SE = 2.98, t = − 12.703, p < 0.001), story (Computer: 378 ms ± 228 ms, Confederate: 353 ms ± 204 ms; M = − 24.91, SE = 2.98, t = − 8.366, p < 0.001), and interval (400 ms Interval: 320 ms ± 190 ms, 700 ms Interval: 411 ms ± 231 ms; M = 90.97, SE = 2.98, t = 30.548, p < 0.001). As in experiment #1, this shows a TB effect for operant and for personal trials and confirms that participants were able to distinguish between long and short durations. Additionally, the factor diagnosis surpassed the significance threshold (TD: 314 ms ± 203 ms, ASD: 414 ms ± 218 ms; M = 99.63, SE = 44.98, t = − 2.215, p = 0.032), showing that, on average, persons with ASD reported longer estimates across all conditions than the TD group. As participants reliably discriminated between 400 and 700 ms durations, we assumed that they paid sufficient attention to the task and the time intervals.

Just as in experiment #1, the interactions between agency and story (M = − 40.70, SE = 5.96, t = − 6.834, p < 0.001) was significant. This confirms social hyperbinding for experiment #2. The interactions story and interval (M = − 12.15, SE = 5.96, t = − 2.040, p = 0.041) and agency and interval (M = − 16.39, SE = 5.96, t = − 2.752, p = 0.006) were significant. In line with the prior experiment and earlier studies^32^, this seems to suggest a floor effect for lower time estimates in TB.

Consistent with this notion, analysis confirmed the interaction between interval and group (M = − 22.17, SE = 5.96, t = − 3.723, p < 0.001) indicating that the group differences in reported estimates were more pronounced for the longer interval (700 ms) than for the shorter interval (400 ms).

**Figure 4:**
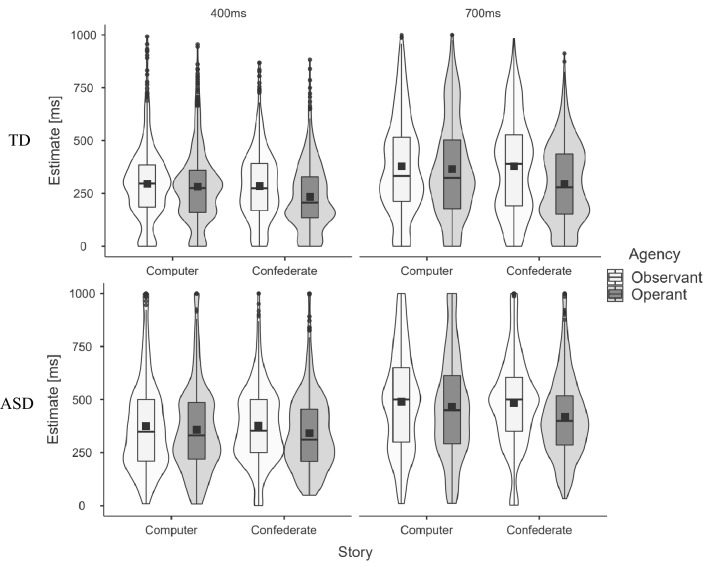
Results for Experiment #2. The top row depicts estimates from the TD group. The bottom row depicts estimates from the ASD group. Plots on the left depict estimates for 400 ms intervals. Plots on the right depict estimates for 700 ms intervals. Time estimates for computer conditions are shown in the left columns; those for confederate conditions are shown in the right columns. Light grey indicates observant conditions, while dark grey indicates operant conditions. Outliers (depicted as black dots) correspond to individual participant’s estimates and reflect grouping around individual anchor points. Social hyperbinding is visible in the relatively lower estimates of operant trials as compared to observant trials for confederate conditions. Hyperbinding is significantly lower for the ASD group.

Concerning group differences—similar to experiment #1—participants in the ASD group showed a less pronounced hyperbinding effect for the Confederate condition resulting in a significant interaction between the factors story and group (M = 16.49, SE = 5.96, t = − 2.768, p = 0.006; see Fig. 4).

Finally, we observed a significant three-way interaction between agency, story, and group (M = 25.40, SE = 11.91, t = − 2.133, p = 0.033; see Fig. 4). Overall, this confirms the hypothesis of a smaller social hyperbinding for the ASD group for social top-down information.

No other interaction reached the significance threshold. We performed post-hoc analyses on additional variables of interest (see Supplement for results and figures). These include a correlation of the effect of interest with autistic trait scores as measured by the AQ and an analysis of within-subjects variance (see Supplement).

As in experiment #1, we did not find a significant correlation between autistic traits and the hyperbinding effect (Table ST2.2a/b). We again propose that future research should clarify whether a lower hyperbinding in ASD reflects a discrete criterium or runs along a continuum of autistic traits.

Our supplementary analyses for changes in participant-wise variance (Fig. SF2.3; Table ST2.1) suggest differences in variance between groups, depending on experimental conditions. This further highlights the importance for future experiments to test for social hyperbinding in the context of other methods assessing time perception in ASD (see our discussion section for a more in-depth discussion of methodological limitations and further research directions).

The supplement further includes figures on the main effects containing further information, such as individual mean estimates and standard deviations (Figs. SF2.1 and 2.2).

#### Discussion for Experiment #2

Experiment #2 was a variation of experiment #1 directed at isolating the differential effect of top-down beliefs on TB. By manipulating participants’ belief in the presence of an interaction partner, we were again able to replicate the overall finding of social hyperbinding in both a group with and a group without ASD. This second replication confirms earlier findings of increased TB during social interaction^32,36,37^ and demonstrates the differential influence of top-down information on this effect.

Concerning differences between individuals with ASD and those without ASD, our results indicate significantly smaller social hyperbinding for persons with ASD, as indicated by the interaction between agency, story, and group. This confirms the initial hypothesis of a less pronounced influence of social information on TB in ASD. Considering the relationship between TB and predictive processing, this finding complements the existing data on differences in predictive processing in ASD.

An interesting and unexpected finding was the observed main effect for group and the seemingly stronger effect of interval duration on time estimation in ASD. In particular, we found generally larger interval estimates and more pronounced effects of the 700 ms interval on the factors partner and agency in the ASD group. One might assume that the group differences reflect a greater accuracy in time perception for the group with ASD in experiment #2. But this interpretation would leave the question unanswered, why participants with ASD were better at judging time in experiment #2 as compared to experiment #1. It is important to note that the method we used in both experiments does not test participants’ ability to estimate time correctly. This would require participants to learn to judge intervals of similar lengths prior to the real task^64,65^. In our opinion, the main effect for group is not readily interpretable as an inherent difference in time perception accuracy between both groups as neither experiment involved any practice trials. Participants were left without feedback on their judgments.

This lack of practice may have led participants to start estimating durations at an arbitrary position on the VAS. This starting point may have served as a random anchor for subsequent estimates. Our mixed effects model accounts for such random interindividual variance with random intercepts for participants. We interpret the interaction effects within and between groups in accordance with our initial hypotheses. However, we cannot sufficiently rule out whether the observed effects were in part influenced by anchoring.

Individuals with ASD show several potential differences in timing and time perception. These differences range from impaired perception of time intervals in both estimation and reproduction tasks (for review see^63,66–68^), over temporal resolution and acuity^14,69–72^, to differences in temporal synchrony (e.g.,^73^) and temporal experience^8,74^. Findings are heterogeneous and as of yet, concerning duration judgments, no specific differences for ASD have been found^68^.

We can speculate that the observed higher accuracy of judgements, or alternatively the relatively higher estimates by individuals with ASD is due to the change of the manipulation, namely, the exclusive use of a geometric, non-facial stimuli. In other words, a face stimulus could have influenced judgments to be more precise or longer. However, this effect of higher accuracy or longer judgments should also be visible in experiment #1 for the non-face stimuli.

Again, our method is insufficient to answer this question reliably. First, the method by Engbert et al.^22,23^ detects judgment biases between conditions but it is explicitly not designed to compare judgements to clock time. Second, we had no prior hypothesis on general differences in time estimation beyond systemic bias between conditions. This is especially the case for experiment #2, which we conducted after experiment #1, which had not yielded such an overall difference.

Lower social binding effect in experiment #2 may be due to higher accuracy in timing. The better time perception of participants with ASD might have masked the relative decrease for the operant condition with a partner. Decreased social hyperbinding in ASD would then be secondary to increased perceptual accuracy.

As an alternative explanation to higher accuracy, we could explain the difference in overall estimates by generally higher internal reference intervals for the group with ASD. The relatively longer memory of one second could have led to larger judgements in general. If this were true, social hyperbinding would not be affected by the overall difference.

Again, our method was not designed to differentiate between these two alternative interpretations. Our finding therefore motivate further investigation into action-event-duration judgements using alternative methods. For example, TB can also be measured using reproduction tasks (e.g.,^75,76^), which might be more suitable to detect true differences in time perception in ASD^68^.

Taken together with the results from experiment #1, we do however believe our results reflect a generally smaller social hyperbinding for the ASD group as compared to the TD group. In experiment #2, this relatively decreased binding was particularly pronounced for the cover story condition as seen in the interaction between story, agency, and group. An overall smaller TB may be related to the strength of event coupling in multisensory cue-integration^20,77^. Accordingly, the relatively smaller TB in the ASD group may correlate to a relatively smaller degree of perceived correlation between voluntary action and partner movement. Conversely, smaller TB may indicate a weaker prediction for social outcomes in participants with ASD, as compared to those without ASD.

### Ethics approval and consent to participate

All procedures performed were in accordance with the ethical standards of the institutional and/or national research committee and with the 1964 Helsinki Declaration and its later amendments or comparable ethical standards. The study was approved by the Ethics Committee of the Medical Faculty of the University of Cologne (No. 17-349). Written informed consent was obtained from all individual participants included in the study. None of the participants were under legal guardianship. All participants were naïve as to the purpose of the experiment. Participants received 10€ per hour as compensation for their participation.

## Discussion

In two sequential experiments, this study employed an established method to assess the temporal binding effect (TB)^22–24^) to investigate predictive processing and the strength of cue integration for social events in a group of participants with autism (ASD) as compared to a group of participants without autism (TD). In an observant condition, participants judged the duration of intervals between a direction cue (arrow) and a subsequent movement on the computer screen. In operant conditions, they judged durations between their voluntary key presses and movements on the computer screen. Movements were either eye movements of a face-like stimulus (experiment #1), or the movement of a dot (experiment #1 and #2). Relatively lower estimates of intervals were interpreted as TB.

Overall results indicate larger TB for events believed to involve another person. While experiment #1 indicates a general alteration in TB for social events, involving both perceptual, bottom-up information, as well as believed, top-down information, experiment #2 clearly demonstrates weaker binding in the ASD group for events involving top-down information on the presence of an interaction partner.

The findings from both experiments replicate earlier findings demonstrating that the mere belief in a human–human interaction suffices to elicit a pronounced binding effect^32^. It further adds to existing information about the privileged processing of social information and its comparable change in processing in individuals with ASD^78,79^. In experiment #1, the introduction of a cover story in combination with a face-like stimulus caused TB for events involving intentional actions with an effect on the behaviour of another person. In experiment #2, a cover story about an allegedly present interaction partner alone produced TB for action-movement causalities.

We were able to extend these earlier findings and implications to individuals with a diagnosis of ASD. Similarly to the group without ASD, participants with the diagnosis showed significant TB for events involving an initializing movement, particularly whenever the action involved a supposed human partner. However, this social TB was significantly less pronounced in the ASD group. On the one hand, the presence of the effect in both groups confirms the general influence of socially relevant top-down information on predictive processing in ASD. On the other hand, its lower expression suggests a substantial difference in how this information is processed.

Our finding could relate to a reduced Sense of Agency (SoA) for actions with an interaction partner. Although differences in SoA appear to exist for individuals with ASD^30,80^ the manipulation of agency was the same in both conditions, social and non-social. We therefore cannot substantiate any speculations on the involvement of SoA on social hyperbinding.

As a hypothetical solution, the initial study by Vogel et al.^32^ had speculated on the origin of the increase in TB for actions involving the belief in another person. In their opinion, social interaction increases event monitoring for reactions to own actions. This improved monitoring capacity in turn increases available information and hence TB. In our opinion, the most likely explanation lies in the prompting of automatic mentalizing processes^32,47^ brought on by the introduction of a confederate. Our findings indicate that both groups most likely initiated the retrieval of social information during confederate trials^38,39,81^. The simulated social interaction promotes the integration of additional top-down social information otherwise not relevant during behaviour in a non-social context. Our results further suggest that increased TB may correlate with this process. As persons with ASD demonstrated a relatively smaller correlation than the TD group could be a hint for a less substantial influence of the social context on time processing in ASD.

This lesser degree of integrated information corresponds to less information involved in the predictive process for social events and a stronger disconnectedness. The level of available information is crucial for an event’s predictability^29^. Arguably, the social information garnered from the cover story would increase information and hence the predictability of the situation. In other words, although the cover story was in itself irrelevant to perform the task, it influenced time judgements. We propose that the initially provided information from the cover story carried over and enhanced TB^32^. Our findings are consistent with the speculation that this subjoined social information is less relevant for predictions in persons with ASD. However, we cannot provide any further insight into the nature of the process, e.g. whether the information from the cover story was less received by persons with ASD, or influenced their TB to a lesser degree. Unfortunately, we did not take measures of mentalizing ability. However, if future research were to validate this theory, social TB might pose a measurable correlate to mentalizing capacities. Furthermore, linking mentalizing with SoA measurements may fill the explanatory gap for the increase in TB for social interaction—and potentially for its quantitative difference for our group with a diagnosis of ASD. In other words, assumptions about the other person might be crucial for establishing a SoJA during interaction.

Considering earlier publications, this study’s gain in knowledge lies in the discovery of a specific predictive impairment in the social domain. Just as the two prior studies on ASD and TB, we do not find a consistent general predictive impairment^30,31^, but there is a significant difference in the degree of involved social information in processing. Taken together, our findings suggest context-sensitive differences in predictive processing for ASD ^82^. This most likely indicates that information processing in ASD is not generally impaired. The difference emerges whenever top-down information needs to be integrated—specifically when this prior information is social information.

Our findings demonstrate the importance of differentiating between behaviour in social and non-social contexts. Currently, insights on time perception in ASD are primarily available for the non-social domain. Our results demonstrate that the content of information may carry additional importance. Therefore, future research on the topic should also include paradigms differentiating between behaviour involving social and non-social information or contexts. We propose that for individuals with ASD, differences in time perception and time related behaviour might be more pronounced for social contexts.

This evidence opens another line of future inquiry. From our results, it is apparent that social information influences cognitive processing as measured by TB. However, we did not directly investigate the added or differential influence of bottom-up perceptual information (e.g., the stimulus’ influence) nor did we test whether other, non-social top-down information might have brought on similar changes to TB and its differences between groups. Several studies have demonstrated effects of social stimuli without a cover story on TB^32,83,84^. In addition, theories exist that claim that processing in ASD is not fundamentally different for social information per se, but for complex and high information loads, of which social information is one particular subtype^4,8,85^. In other words, despite being able to confirm our hypothesis on a social belief’s influence on TB in ASD, we cannot answer the question whether we would find similar effects for social stimuli without belief manipulation, or for other added, non-social beliefs about the interactive situation.

## Limitations

There are several caveats and limitations to our studies. First, as stated in the discussion section, we did not determine or measure mentalizing during our experiments, nor did we ascertain mentalizing capacity in general. This makes some of our assumptions on the roots of the observed effect speculative. Future research and improved paradigms should address this issue. Second, the two groups in both studies were primarily matched according to demographic variables. This leaves out potentially confounding variables such as e.g., IQ. Third, we found several differences in time perception, which our method is not fully suitable to explain. Additional investigations using alternative paradigms, such as e.g., time reproduction tasks, are necessary to investigate these potential differences. Fourth, we did not employ techniques to objectivize attention and understanding of instructions. This may explain some of the more extreme judgements on the edges of the VAS. Future replications of social hyperbinding should include, e.g., catch trials or response-based exclusion criteria to better account for these sources of variance. The same may be true for potential bias introduced by using a VAS without prior practice trials. Prior time estimation training establishes clock time measurements as anchors^86,87^. This allows for better control of interindividual variance. Lastly, more research and in particular thorough replication study will be necessary to guarantee generalizability across the ASD spectrum. Although our results clearly replicate the social hyperbinding effect for individuals without a psychiatric diagnosis, replicability will have to be proven for the group with ASD.

## Conclusions

This study adds to the evidence of a predictive processing difference for individuals with ASD in the social domain^2,3^. It replicates existing experimental data on a social hyperbinding effect^32^ and action event monitoring during social interaction^88,89^. The knowledge gained motivates future research questions on predictive processing, SoA, and mentalizing in ASD.

## Supplementary Information

Supplementary Information.

## Data Availability

The datasets used and/or analyzed during the current study are available from the corresponding author on reasonable request.

## Abbreviations

ASD: Autism Spectrum Disorder
TB: Temporal binding
SoA: Sense of agency
AQ: Autism quotient
SE: Standard error
