# Supplementary material for: Temporal binding of social events less pronounced in individuals with Autism Spectrum Disorder
Source: Sci Rep. 2022 Sep 1;12:14853. doi: 10.1038/s41598-022-19309-y (PMC9437002; doi:10.1038/s41598-022-19309-y)
Supplement: Supplementary file 1 — Supplementary Information. [file 41598_2022_19309_MOESM1_ESM.docx]

**Supplement:**

S1 Supplement for experiment #1:

Figure SF1.1: Simplified depiction of effects as mean intervals with standard error bars according to O’Brien & Cousineau [1].


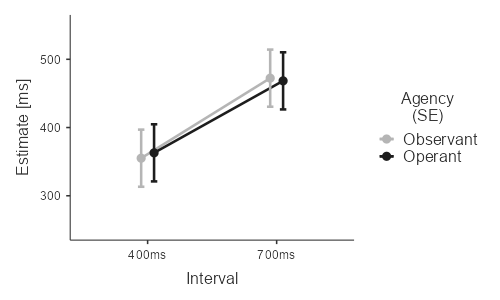

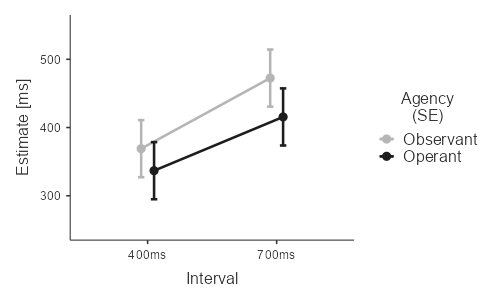


Physical

Personal

TD

ASD


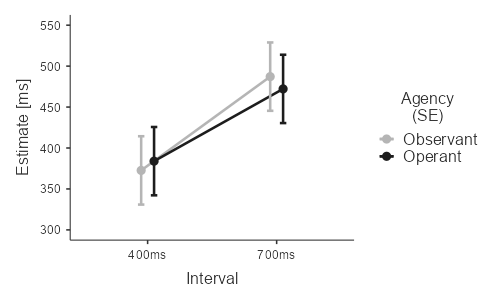

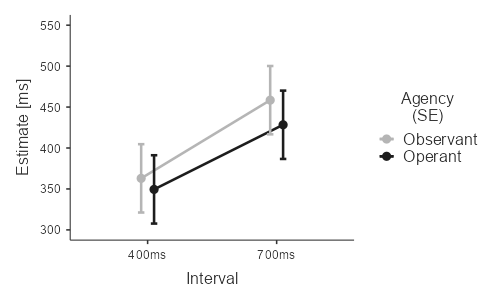


Figure SF1.2: Simplified depiction of effects as mean intervals with standard error bars according to O’Brien & Cousineau [1]. The plots include the individual mean estimates of each participant. The plots visualize the broad range of mean estimates. These means may reflect interindividual variance caused by a random anchoring effect.

Physical

Personal

TD

ASD


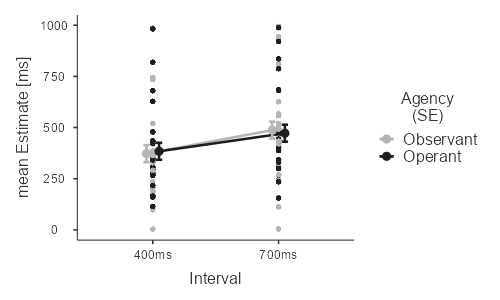

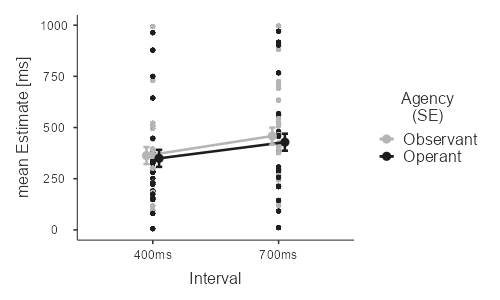

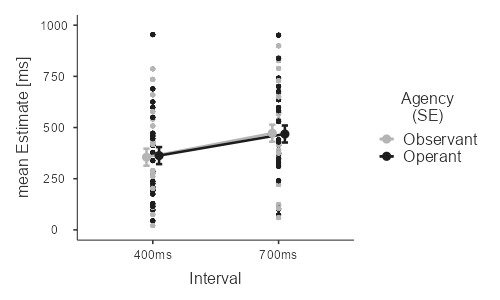

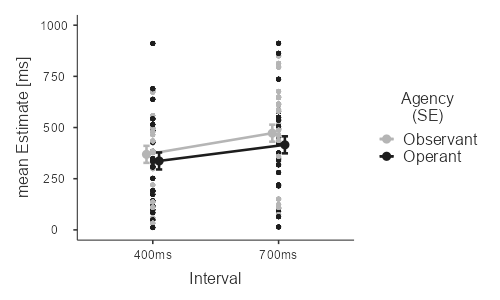


Figure SF1.3: Intraindividual Standard Deviations plot. The plots depict the mean taken from the individual standard deviations of each participant. They reflect the variance of within-subject variance across conditions. Overall, intraindividual variance increases with interval duration. However, its also shows distinct differences in variance between groups and conditions, reflecting corresponding differences in estimate precision.


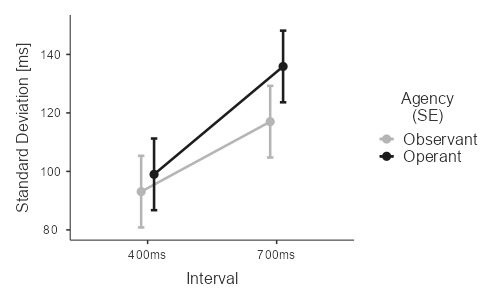

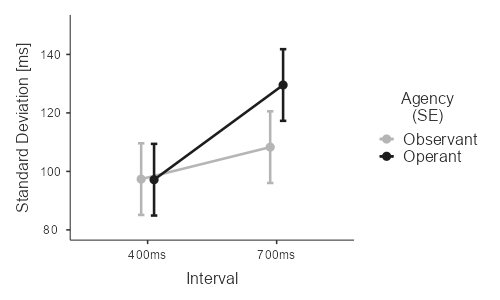


Physical

Personal

TD

ASD


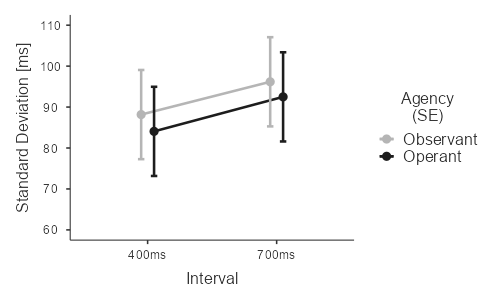

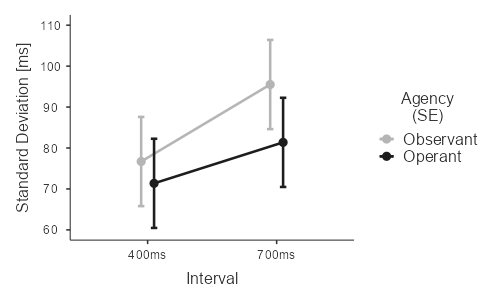


Table ST1.1: GLM of individual standard deviations dependent on mean estimates by diagnosis. The model reveals the increase of the mean within-subject variance depending on increasing mean time estimates. This finding can be interpreted as an overall adherence of the data to scalar expectancy theory (Allman and Meck, 2012). There was no significant difference in this effect between diagnostic groups.


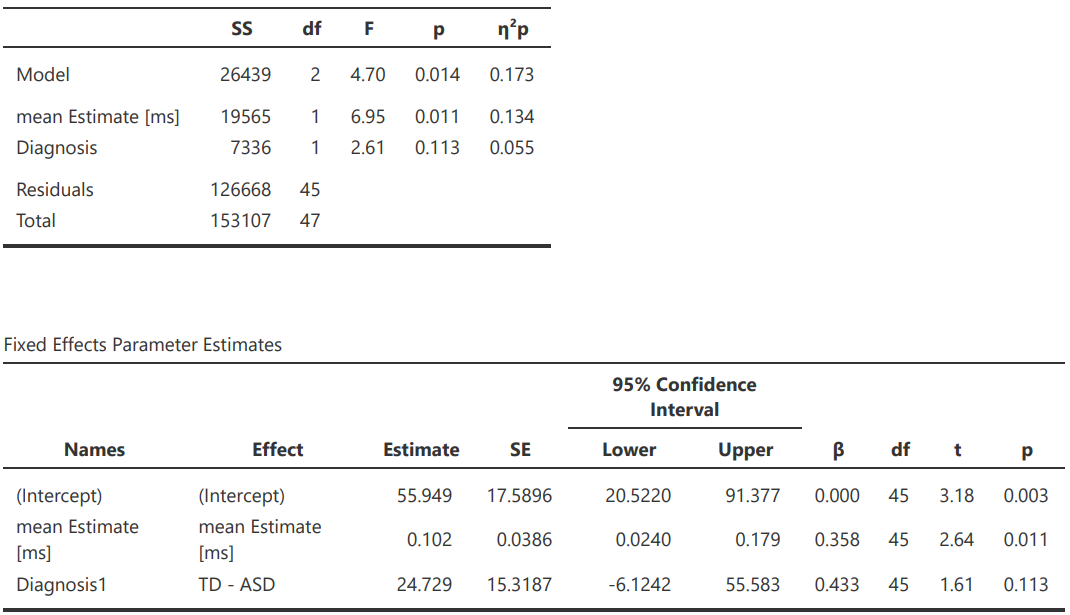


Figure SF1.4: Correlation between individual standard deviations and mean estimates. The plot depicts the results from table ST1.1 and the assumption of the data’s adherence to scalar expectancy theory (Allman and Meck, 2012).


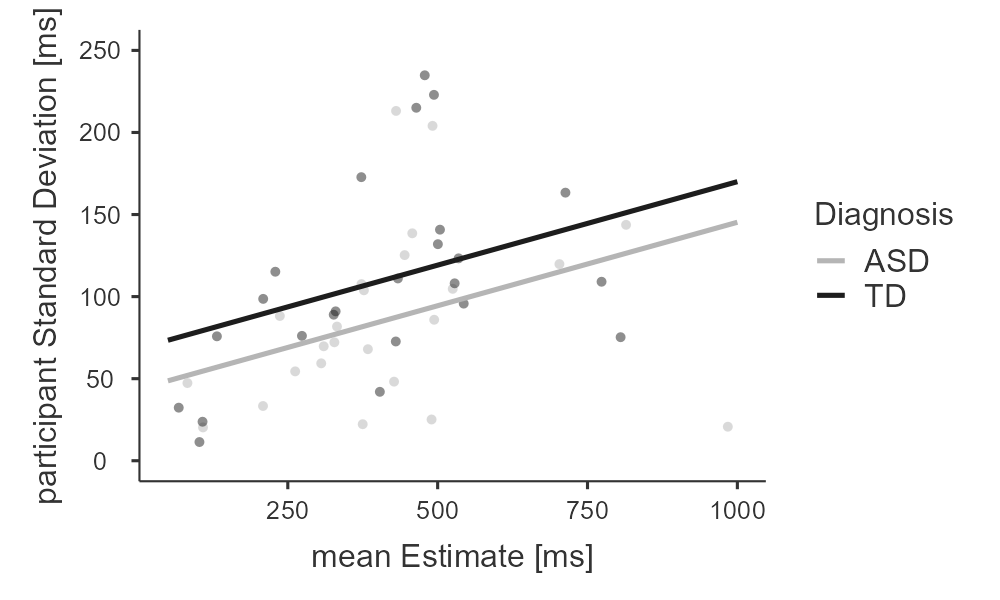


Table ST1.2a: Correlation between AQ-scores and social hyperbinding (mean estimates for personal observant conditions - mean estimates for personal operant conditions) for 400ms intervals. In neither diagnostic group, we were able to detect a significant correlation of social hyperbinding with AQ scores for medium sub-second intervals. Future research should clarify whether a lower hyperbinding in ASD reflects a discrete criterium potentially reflecting a discrete condition, or, whether it runs along a continuum of autistic traits corresponding to a continuum of traits between persons with autism and persons without autism.


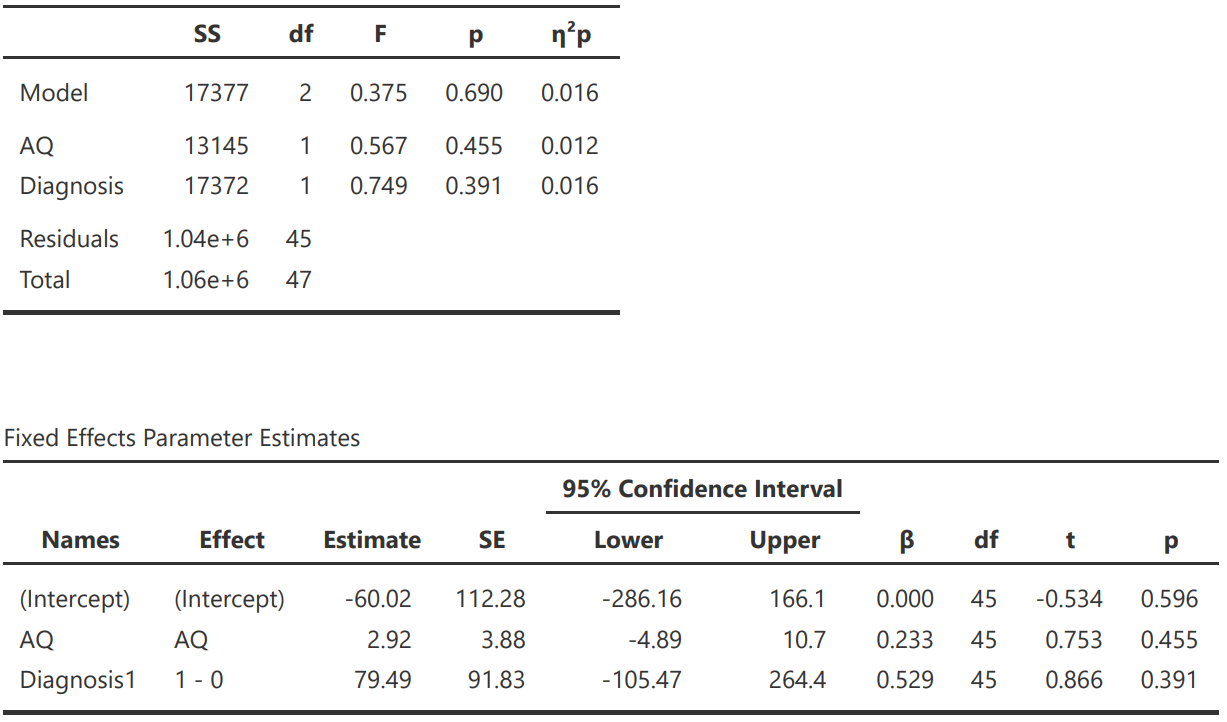


Table ST1.2b: Correlation between AQ-scores and social hyperbinding (mean estimates for personal observant conditions - mean estimates for personal operant conditions) for 700ms intervals. In neither diagnostic group, we were able to detect a significant correlation of social hyperbinding with AQ scores for larger sub-second intervals. Future research should clarify whether a lower hyperbinding in ASD reflects a discrete criterium potentially reflecting a discrete condition, or, whether it runs along a continuum of autistic traits corresponding to a continuum of traits between persons with autism and persons without autism.


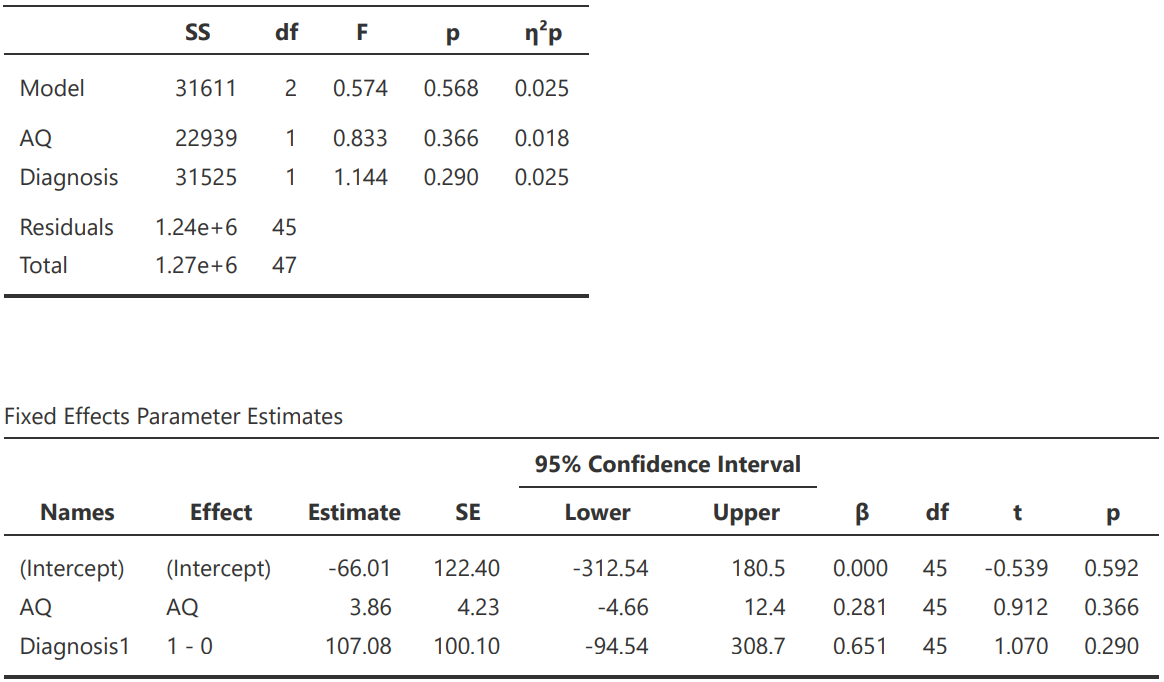


S2 Supplement for experiment #2:

Figure SF2.1: Simplified depiction of effects as mean intervals with standard error bars according to O’Brien & Cousineau [1].

400ms

700ms

TD

ASD


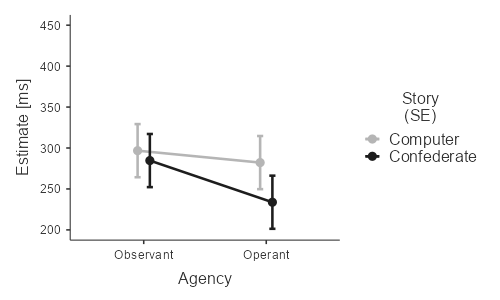

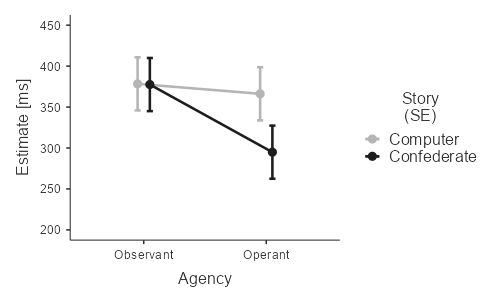

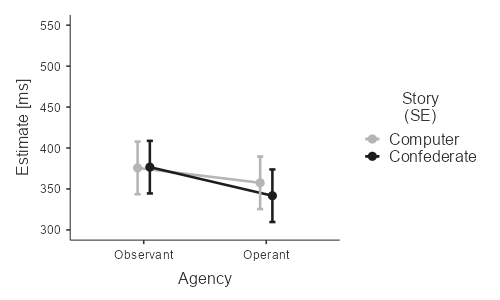

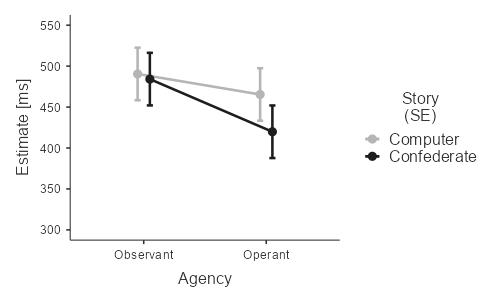


Figure SF2.2: Simplified depiction of effects as mean intervals with standard error bars according to O’Brien & Cousineau [1]. The plots include the individual mean estimates of each participant. The plots visualize the broad range of mean estimates. These means may reflect interindividual variance caused by a random anchoring effect.

400ms

700ms

TD

ASD


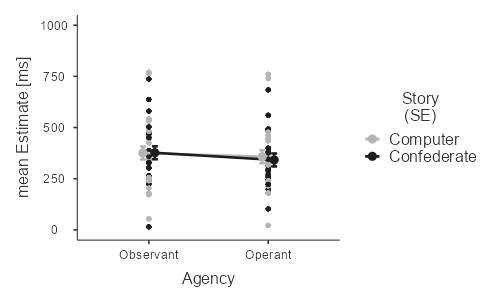

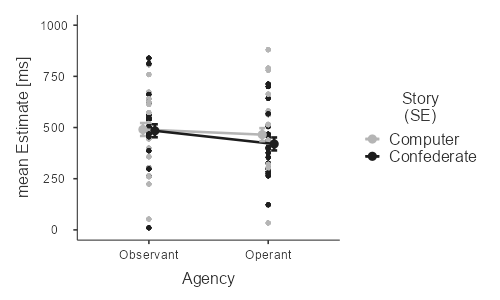

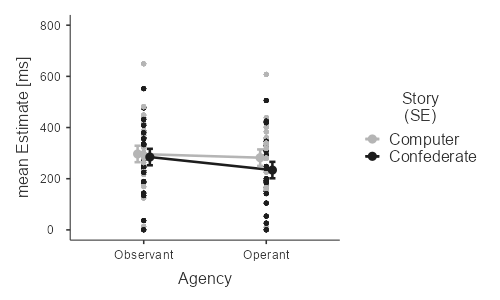

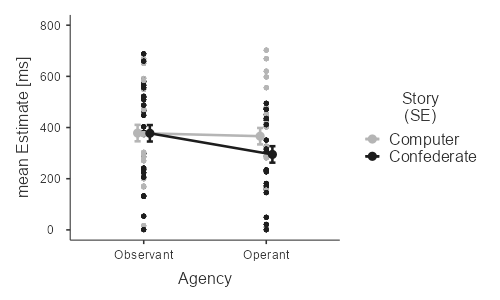


Figure SF2.3: Intraindividual Standard Deviations plot. The plots depict the mean taken from the individual standard deviations of each participant. They reflect the variance of within-subject variance across conditions. Overall, intraindividual variance increases with interval duration. However, it also shows distinct differences in variance between groups and conditions, reflecting corresponding differences in estimate precision.

400ms

700ms

TD

ASD


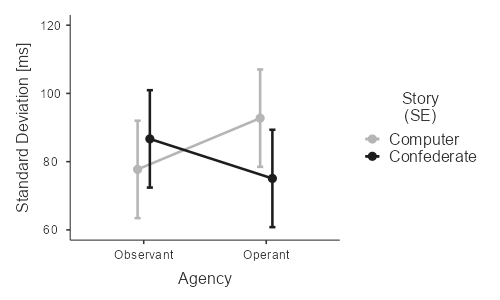

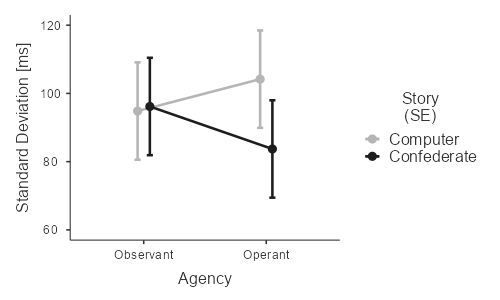

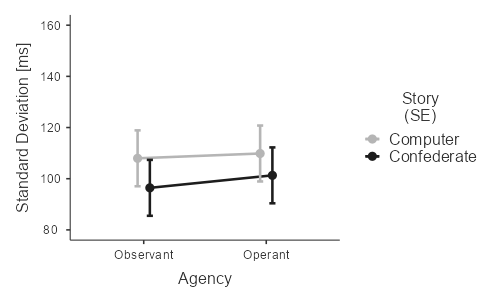

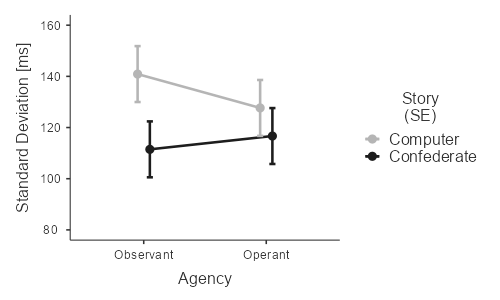


Table ST2.1: GLM of individual standard deviations dependent on mean estimates by diagnosis. The model reveals the increase of the mean within-subject variance depending on increasing mean time estimates. This finding can be interpreted as an overall adherence of the data to scalar expectancy theory (Allman and Meck, 2012). There was no significant difference in this effect between diagnostic groups.


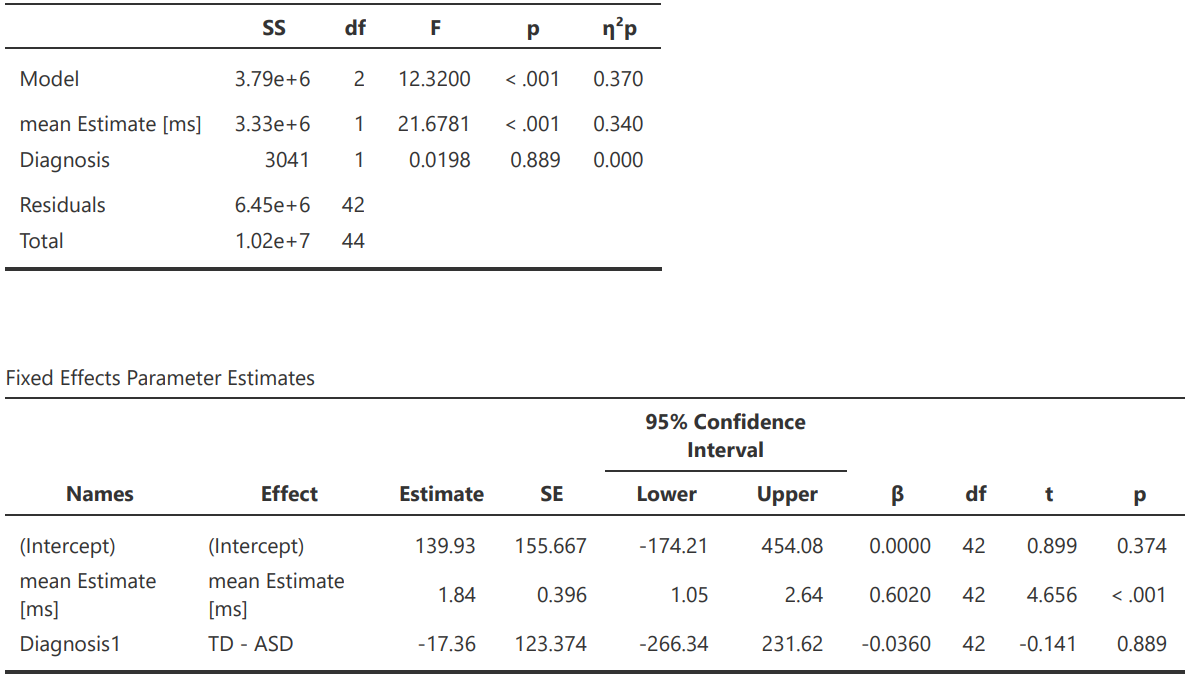


Figure SF2.4: Correlation between individual standard deviations and mean estimates. The plot depicts the results from table ST1.1 and the assumption of the data’s adherence to scalar expectancy theory (Allman and Meck, 2012) with no discernable difference between groups. One participant excibitted estimates including and very close to 0ms. Their estimates ranged between 0 and 2ms. The mean estimate for 400ms intervals was 0.31ms; for 700ms was 0.73ms; and in total was 0.52ms. The participant still showed sufficient discrimination from 0ms (one-sided Student’s T-test against 0ms p<0.001) and was able to differentiate significantly between 400ms and 700ms intervals (one sided Student’s T-test p<0.001).


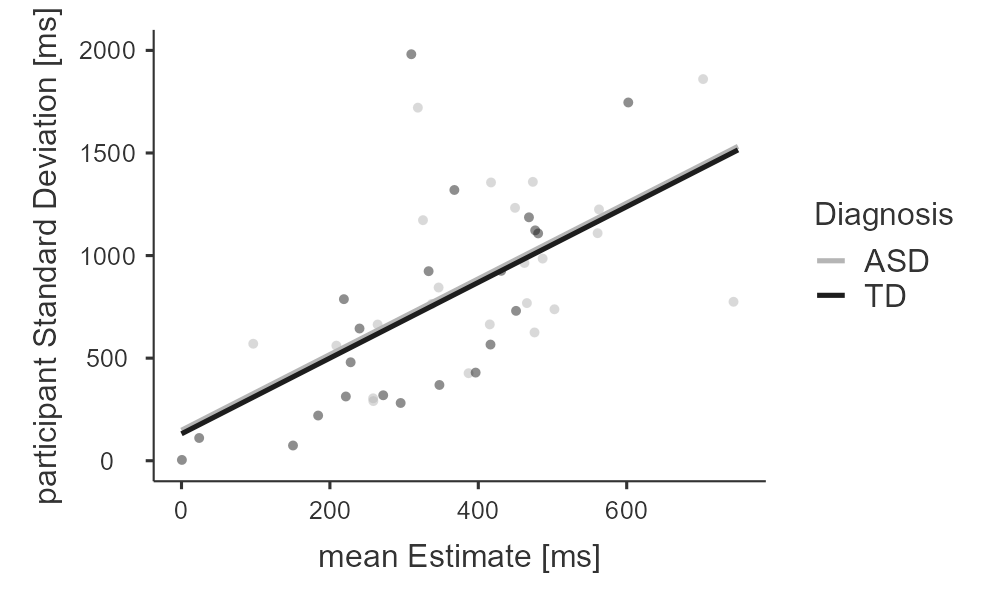


Table ST2.2a: Correlation between AQ-scores and social hyperbinding (mean estimates for personal observant conditions - mean estimates for personal operant conditions) for 400ms intervals. . In neither diagnostic group, we were able to detect a significant correlation of social hyperbinding with AQ scores for medium sub-second intervals. Future research should clarify whether a lower hyperbinding in ASD reflects a discrete criterium potentially reflecting a discrete condition, or, whether it runs along a continuum of autistic traits corresponding to a continuum of traits between persons with autism and persons without autism.


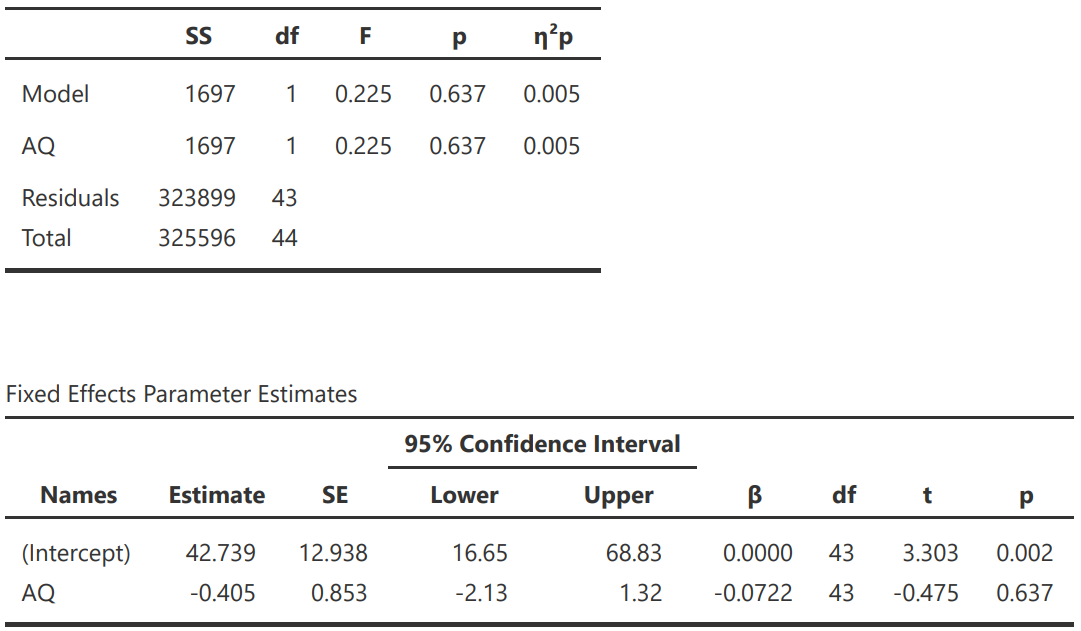


Table ST2.2b: Correlation between AQ-scores and social hyperbinding (mean estimates for personal observant conditions - mean estimates for personal operant conditions) for 700ms intervals. . In neither diagnostic group, we were able to detect a significant correlation of social hyperbinding with AQ scores for larger sub-second intervals. Future research should clarify whether a lower hyperbinding in ASD reflects a discrete criterium potentially reflecting a discrete condition, or, whether it runs along a continuum of autistic traits corresponding to a continuum of traits between persons with autism and persons without autism.


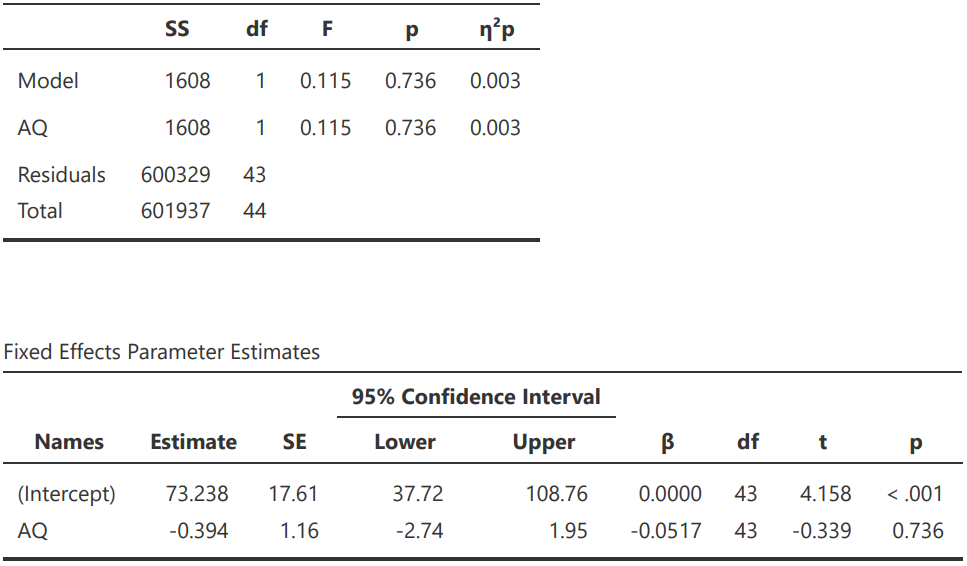


**Literature:**

1 O’Brien, F., & Cousineau, D. (2014). Representing error bars in within-subject designs in typical software packages. The Quantitative Methods for Psyschology, 10(1), 56–67.
